# Barriers and enablers in the implementation and sustainability of toothbrushing programs in early childhood settings and primary schools: a systematic review

**DOI:** 10.1186/s12903-022-02270-7

**Published:** 2022-06-18

**Authors:** Navira Chandio, Sowbhagya Micheal, Santosh Kumar Tadakmadla, Woosung Sohn, Susan Cartwright, Rhiannon White, Prathyusha Sanagavarapu, Jinal Shashin Parmar, Amit Arora

**Affiliations:** 1 School of Medicine, Western Sydney University, Campbelltown Campus, Locked Bag 1797, Penrith, NSW 2751 Australia; 2 Health Equity Laboratory, Campbelltown, NSW 2560 Australia; 3 School of Health Sciences, Western Sydney University, Campbelltown Campus, Locked Bag 1797, Penrith, NSW 2751 Australia; 4 Translational Health Research Institute, Western Sydney University, Campbelltown Campus, Locked Bag 1797, Penrith, NSW 2751 Australia; 5 Department of Rural Clinical Sciences, Violet Vines Centre for Rural Health Research, La Trobe Rural Health School, Bendigo, VIC 3550 Australia; 6 Sydney Dental School, Faculty of Medicine and Health, The University of Sydney, Surry Hills, 2010 Australia; 7 Colgate-Palmolive Pty Ltd., 345 George St., Sydney, 2001 Australia; 8 School of Education, Western Sydney University, Bankstown Campus, Locked Bag 1797, Penrith, NSW 2751 Australia; 9 Oral Health Services, Sydney Local Health District and Sydney Dental Hospital, NSW Health, Surry Hills, NSW 2010 Australia; 10 Discipline of Child and Adolescent Health, Sydney Medical School, Faculty of Medicine and Health, The University of Sydney, Westmead, NSW 2145 Australia

**Keywords:** Child, Preschool, Schools, Daycare, Oral hygiene, Toothbrushing, Dental caries, Enablers, Barriers

## Abstract

**Background:**

Untreated dental caries negatively impacts a child's quality of life including overall health and wellbeing, growth and development, social interaction ability, and school attendance. School-based toothbrushing programs have been recognised as an effective intervention to reduce the burden of dental caries. However, limited information is available to understand the real-world enablers and challenges in the implementation and sustainability of toothbrushing programs. This review aims to understand the barriers and enablers in the implementation and sustainability of toothbrushing programs in early childhood settings and primary schools.

**Methods:**

Five electronic databases [i.e., CINAHL (EBSCO), Medline (EBSCO), EMBASE (Ovid), Web of Science, and PsycINFO] and backward citation chasing were performed. The last updated databases searches were conducted in May 2022. Studies reporting on barriers and enablers in the implementation and sustainability of toothbrushing programs in early childhood settings or primary schools were included in the review. The methodological quality of included studies was assessed by using Joanna Briggs Institute [JBI] and mixed methods appraisal tool [MMAT] critical appraisal tools and results were reported in accordance with PRISMA guidelines.

**Results:**

A total of six studies met the eligibility criteria and were included in the review. Toothbrushing programs in early childhood settings and primary schools were mostly implemented under the supervision of staff and teachers. A positive attitude of the staff, the flexibility of toothbrushing sessions, involvement of community volunteers and parents were a few of the identified enablers. However, the timing of the communication of the program, inadequate transfer of information among staff, frequent staffing turnover, lack of parental support, and staff feeling overburdened while acting as pseudo parents were frequently reported as barriers.

**Conclusion:**

The results of this systematic review identify key enablers and barriers for toothbrushing programs in early childhood settings and primary schools which need to be considered for developing oral health promotion initiatives.

**Supplementary Information:**

The online version contains supplementary material available at 10.1186/s12903-022-02270-7.

## Introduction

Dental caries is one of the most prevalent non-communicable diseases of global public health concern affecting children [1]. The Global Burden of Disease study (2017) has reported an estimated 530 million children had tooth decay in their primary teeth [2]. In Australia, dental caries is recognised as a non-fatal burden of oral disease and significantly affects children aged 5–14 years [3]. According to the Australian National Child Oral Health Study, more than 25% of children aged 5–10 years had untreated dental caries in their primary teeth, and approximately one in ten children aged 6–10 years had dental caries in the permanent teeth [4]. This problem of dental caries is not unique to Australia. In the United Kingdom (UK), the national child dental health surveys have reported limited change in the prevalence of dental caries over the period of the last 20 years among five-year-old children [5]. In 2019, the Oral Health Surveillance Report from the United States of America concluded little change in the prevalence of dental caries among 2–8-year-old children from 1999–2004 and 2011–2016 [6]. Untreated dental caries impacts a child's quality of life including overall health and wellbeing, growth and development, social interaction, and poor school attendance [7, 8].

Dental caries has a multifactorial etiology with key factors such as the frequent consumption of sugary diet, inadequate exposure to fluoride, and inadequate toothbrushing [9]. Moreover, social determinants of poor oral health such as deprived socioeconomic status increase the risk of dental caries in children [10, 11]. Despite the high burden of dental caries in children, it is worthy to note that dental caries is preventable [12]. Effective preventive measures for dental caries in children require the adoption of oral health promoting behaviours such as a low sugar diet, twice-daily toothbrushing with age-appropriate fluoridated toothpaste, and regular dental visits [9].

Childhood and adolescence are the most influential stages of developing healthy behaviours [13]. The oral health-related attitudes, beliefs, and behaviours developed this period will potentially be sustained throughout life [13]. Apart from fluoridated water, toothbrushing with a fluoride toothpaste is one of the most readily available form of fluoride [14] and twice daily toothbrushing with a fluoride toothpaste has proven to be effective in preventing caries [15]. Therefore, several toothbrushing programs have been implemented in early childhood settings (such as day-cares and pre-schools) and primary schools to reduce the burden of oral health disparities in children. For example, a once daily school-based toothbrushing program in London showed the overall caries increment in the interventional group (2.60) was significantly less (*p* < 0.001) than children in the control group without intervention (2.92) [16]. Likewise, a toothbrushing educational programs in preschools demonstrated significant reduction in plague and caries development among children in experimental group than controls (non-intervention groups) [17]. It is worth noting toothbrushing programs are not unique to high-income countries. For instance, a school-based oral health promotional program in Uganda revealed remarkable improvement in school children's oral health by reporting fewer incidents of dental pain, emergency dental visits and significant improvement in their school attendance [18].

Several systematic reviews have been undertaken to assess oral health interventions in early childhood and school settings; however, the none of the reviews explain the barriers and enablers in the implementation and sustainability of the toothbrushing programs [19–22]. Most of these reviews draw evidence from randomised controlled trials (RCT) [20, 21] and quasi-RCTs [19] except for scoping reviews [22] which draw evidence from international guidelines and toothbrushing programs. The outcomes of these reviews were based on the effectiveness of oral health or toothbrushing programs on the incidence of dental caries [19, 21] or improvement in oral hygiene [20, 21]. Most of these reviews searched the evidence for both children and adolescents and did not distinguish research findings specifically for children alone [19, 21]. Moreover, all these reviews rated “critical low” when their methodological quality appraisal was conducted by using AMSTAR 2 tool (see Additional file 1: Appendix 1). The limitation in these previously published reviews demands the need for a high-quality systematic review to identify barriers and enablers in the implementation and sustainability of toothbrushing programs in early childhood settings and primary schools.

The term implementation is defined as for the improvement of the population health, the utilisation of strategies for the change or the introduction of evidence-based health interventions (EBIs) within targeted settings [23]. An evaluation of these adopted and used EBIs strategies in specific settings such as schools, healthcare facilities, or workplaces for the population health sustainability is known as implementation science (IS) [24]. In addition to assessing the effectiveness of school-based oral health program’s [19, 21], it is imperative to understand the enablers and challenges of program implementation for the optimisation of program benefits, sustainability, and dissemination of the findings to other settings [25]. The Implementation research utilises variety of frameworks such as the consolidated framework for implementation research (CFIR), exploration, preparation, implementation, and sustainment (EPIS), reach, effectiveness, adoption, implementation, and maintenance (RE-AIM), and practical, robust, implementation sustainability model (PRISM) [26]. Among other IS frameworks, the utilisation CFIR is the most robust strategy to understand the school-based toothbrushing programs enablers and challenges, because CFIR is a meta-framework and combines elements from other IR frameworks, making it more comprehensive [27]. Moreover, CFIR is widely used worldwide for process evaluation, which relays on understanding how the intervention is implemented and the factors influencing the implementation of intervention [26].The CFIR suggests the implementation is influenced by the intervention characteristics (e.g., implementation decisions, evidence, adaptability, relative advantage, design quality, complexity, and cost), inner settings (e.g., organisational structure, culture, networks, and communications, implementation environment and readiness for implementation), outer settings (e.g., participants needs, cosmopolitanism, external policy, peer pressure, and incentives), the stakeholders involved (e.g., knowledge and belief related to intervention, implementation skills, self-efficacy, personal attributes and identification with the organisation) and the implementation process (e.g., coordination, engagement, execution to plan, reflection and evaluation) [27]. This framework highlights that implementation is a critical process between the decision of an organisation to adopt an intervention and the willingness of the stakeholders to utilise the intervention in routine. Although the Consolidated Framework for Implementation Research [25] is useful, limited evidence exists on the implementation of oral health interventions such as toothbrushing programs in early childhood settings and primary schools. Therefore, the objective of this systematic review is to understand the barriers and enablers to the implementation and sustainability of toothbrushing programs in early childhood settings (such as day care and preschools) and primary schools.

## Methods

This review has been reported according to the preferred reporting items for systematic reviews and meta-analysis (PRISMA) guidelines [28]. The protocol of this systematic review has been registered and published with the PROSPERO International Prospective Register of Systematic Reviews (CRD42022312080) [29]. Initially, the eligibility criteria of the systematic review protocol only included the oral health promotional studies conducted in preschool and primary school settings. Later, the amendment was made and early childhood settings were also added as a part of review eligibility criteria.

### Eligibility criteria

All studies that met the following criteria were eligible for inclusion in this review: (1) studies conducted in early childhood settings and primary schools targeting healthy children (aged 0–13 years), (2) studies reporting on oral health programs that included toothbrushing with a fluoridated toothpaste as an essential component, (3) studies reporting on enablers or barriers in the implementation of toothbrushing programs, (4) full-text articles available in the English language. The inclusion and exclusion criteria for this systematic review are included in Additional file 2: Appendix 2.

### Information sources

Five electronic databases were searched for this systematic review: MEDLINE (OVID), Embase (OVID), Cumulative Index to Nursing and Allied Health Literature (CINAHL) (EBSCO), PsycINFO, and Web of Science (ISI). These databases were searched without any restriction on publication date (i.e., from the time of inception to present), type or region. Additionally, a backward citation chasing (reference lists of included studies) was performed to include all relevant research evidence on the topic.

### Search strategy

The Population Intervention Comparator Outcome (PICO) criteria was used to devise the review question and relevant search terms (Additional file 3: Appendix 3). A combination of specific medical subject headings (MESH) terms and text words were drafted in consultation with a Health Sciences librarian. The search strategy was pre-tested in MEDLINE (OVID) database and subsequently adapted for four other databases. To narrow down or widen the search scope, Boolean operators ‘AND’ and ‘OR’ were used. The search was conducted until 1 May 2021 and then updated until 17th May 2022. The search strategies of all databases are presented in Additional file 4: Appendix 4.

### Study selection process

Studies identified through the electronic databases and citation chasing were uploaded into a reference manager software Endnote 20 (Clarivate Analytics, USA) [30] for removing duplicates, screening, and selection. Two reviewers (NC and AA) independently and in duplicate assessed the title and abstracts of the articles and determined whether the articles met the eligibility criteria (See Additional file 2: Appendix 2). All studies that met the inclusion criteria were retrieved in full text and details of these studies were imported into the JBI System for the Unified Management, Assessment and Review of Information (JBI SUMARI) (Joanna Briggs Institute, Adelaide, Australia). Any studies that had uncertainty regarding the eligibility, were also retrieved in full-text and for the additional information and the study authors contacted. A total of three contact attempts with the authors of the article were made, and in case of no response, based on available information the article was screened. Any disagreements were resolved through discussion including a third reviewer (SM). The reasons for excluding studies after reading full-text articles are reported in Additional file 5: Appendix 5. The process of study selection was carried out in accordance with the PRISMA checklist (Additional file 6: Appendix 6) and presented as a flow diagram.

### Data collection process and data items

A standardised data extraction form was developed based on the checklist provided by the Cochrane Handbook for Systematic Reviews of Interventions [31]. The form was calibrated and pilot-tested by extracting relevant information from two studies to ensure consistency across reviewers and capturing all relevant data. Data from all the included studies were independently extracted by three reviewers (NC, JP, and AA). The following information was extracted from each included study: title, first author, year of publication, study design, study setting, study participants information, data collection methodology, detailed information of oral health program, study outcomes, and funding source.

### Quality assessment

Three reviewers (SC, RW and PS) independently conducted a quality assessment of selected studies by using MMAT [32] for mixed-method studies and JBI Critical Appraisal tools [33] for qualitative and cross-sectional studies. The MMAT tool consists of eight questions for the methodology assessment of a study. The JBI tool for qualitative studies is a 10-item instrument and that of cross-sectional studies is a nine-item instrument. The results of the MMAT and JBI quality assessments were reported narratively by indicating the methodological issues and how these may influence the interpretation of the results. All studies were included in this review irrespective of methodological quality. Disagreements were resolved through discussion with the other reviewer (ST, WS).

### Data synthesis

In this review, the included studies were descriptive cross-sectional, qualitative, and mixed-method studies. For cross-sectional studies, data on enablers and barriers were reported descriptively by presenting frequencies and percentages. The association between parental knowledge and attitude towards toothbrushing programs was reported by analysing the Chi-square test at a 5% level of significance. The Pearson correlation coefficient was reported (if data was available) to explain the relationship between participants' willingness for the sustainability of the toothbrushing program with its predicting factors. A thematic synthesis approach was utilised to report the barriers and enablers information in the qualitative studies. Likewise, for mixed-method studies, the survey results on enablers and barriers were reported descriptively (mean, standard deviation, frequency, and percentage), and a thematic synthesis approach was utilised to synthesis qualitative information.

The initial analysis of qualitative and quantitative studies was conducted by three reviewers independently (NC, JP and AA). In the first step, the reviewer summarised the results by coding barriers and enablers extracted from the quantitative data. Then, the extracted qualitative data from each study were coded to develop themes and subthemes. For each identified subtheme, the coded data were categorised as "barrier" or "enabler". In the second step, the fourth reviewer (SM) read the initial draft of emerging themes and descriptions, to ensure the trustworthiness of the extracted data. At the final step, from the descriptive themes, the analytical themes were developed with the consensus of all the reviewers.

The meta-analysis of the included studies was not possible, due to the low number of included studies and the utilisation of diversified methodological approaches and outcomes in the studies.

## Results

**Fig. 1:**
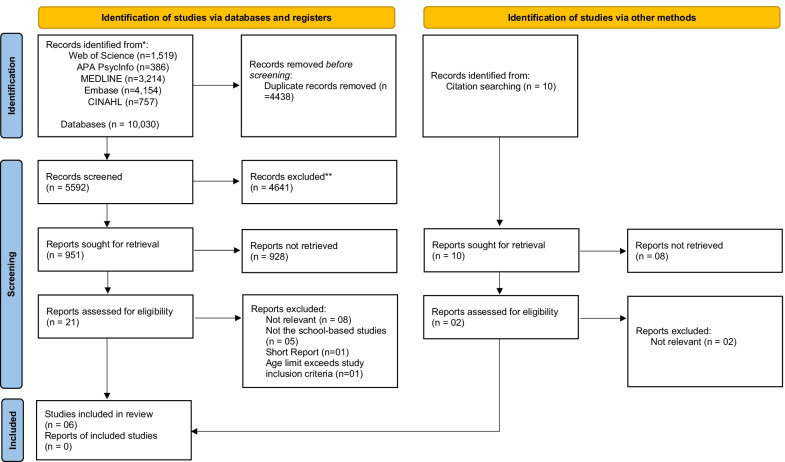
PRISMA flow diagram showing identification of studies via databases

A total of 10,040 studies were retrieved from the five electronic databases (n = 10,030) and citation chasing (n = 10). After the removal of duplicates, 5592 studies were retained for further reading. Of these, 4641 studies were excluded after reviewing the titles and abstracts of the studies and a total of 23 full-text studies were retrieved for reading. Of these, six studies were included in the review. The excluded studies (n = 17) and the reasons for exclusion are summarised in Additional file 5: Appendix 5. The PRISMA flow diagram shows the identification, screening, eligibility, and included studies in Fig. 1.

### Study characteristics

A total of six studies were included in this systematic review [34–39]. Two studies adopted qualitative methodology [34, 35], two studies were cross-sectional studies [36, 38], and the remaining two employed a mixed-methods approach [37, 39]. The characteristics of all the included studies are presented in Tables 1 and 2. The included studies were conducted in Australia [34], the UK [35, 37], Israel [38], Switzerland [36], and Tanzania [39]. Three studies were conducted in early childhood settings [36–38], and three in primary school settings [34, 35, 39]. All studies focused on toothbrushing programs targeting children aged 3–12 years [34–39].

**Table 1:** Characteristics of included studies

Author, year, (country)	Study aims	Study setting and methods	Participants	Outcome		Limitations	Funding source
				Enablers	Barriers		
Dimitropoulos et al. (Australia) [30]	Possible challenges and barriers in continuation of school tooth brushing program	Primary schools; Focus groups	School staff and oral health aide	*School staff level* -Positive attitude, acceptability, and adaptability *Organisational level* -Local school staff and oral health aides infection control training, -Classroom-based toothbrushing activities, -Authoritative school staff, -Strong local school leadership, ***-***Peer support -Flexibility of program implementation timings *Children level* -Program acceptability and children acceptance of lunchtime tooth brushing	*Organisational level* -Whole school toothbrushing activity *School-level* -In-cooperation of the program in school daily routine (initial concern) *Children level* -Older age children program acceptability issues, -Resistance of early morning toothbrushing -Mishandling of toothpaste	-One school did not consent for focus groups -Teacher’s response influenced by program supportive environment -Increased local community participation (community collaborative implementation approach)	Not given
Yusuf et al. (England) [31]	Identifying barriers and enablers in fluoride varnish and toothbrushing programs implementation	Primary School; semi-structured interviews	Health champions (volunteers), general dental practitioners, and school staff	*Children level* -Children’s participation in toothbrushing activities (79.2%) *Schools level* -Adaptation of various parental consent approaches -Improve parents program engagement with the assistance of health champions (program Somali community volunteers’) -Program protocol development aimed at schools for facilitation of implementation -Program implementation flexible timelines -Adequate sharing of information among school staff -Program information translation in Arabic and Somali languages for parents *School-level* -Acceptability of health-promoting schools and by volunteers (Health Champions) and the dental team	*School-level* **-**Some schools struggle with the return of the consent forms from parents **-**Program communication issues with schools were highlighted by school staff **-**Inadequate transfer of information from head staff to the school staff *School staff* -Frustrated due to internal organizational factors, time, and space issues	-It was a pilot study and results cannot be generalised to a wider population	Not specified
Glaser-Ammann et al. (Switzerland) [32]	Parents knowledge and attitude towards school dental health programs	Early childhood setting, questionnaire-based surveys	Parents-children’s dyads	*Parents level* -72% of parents accepted the importance of school dental programs in preschools -72% attended the prophylaxis programs -One fourth (25%) of the parents reported the dental health instructor as the best teacher for children toothbrushing learning skills -One fifth (20%) believe school dental instructor is also the right person to teach a healthy diet -Parents of children who were caries-free were more intended to participate in school dental programs (*p* = 0.11) -The statistically non-significant association was observed between parents' attendance in school dental health program and their educational level (*p* = 0.11), country of origin (*p* = 0.07), and their income (*p* = 0.07) *Children level* -60% believes that their child has benefitted from the program and now brush their teeth better -Just 36% reported that their child consumed healthy mid-morning snacks after the school dental health programs	*Parents level* -Parents assumed that the kindergarten teacher’s role in teaching toothbrushing skills is not important	-Study design limits the study statistical analysis to be considered explorative, and regression analysis and Bonferroni corrections were performed	Not given
Woodall et al. (UK) [33]	Toothbrushing intervention effectiveness and process issues related to its coordination and delivery	Early childhood setting; case studies, interviews, surveys	Parents, children, school staff, oral health promotors	*School staff level* -Acceptability of the program *School-level* -Role of teaching support workers as the main contact point of program co-ordination with oral health promoters, -Linking toothbrushing intervention with school educational curriculum, -Training of school staff, -Provision of adequate information to parents along with children's weekly oral hygiene updates *Parental level* **-**Program acceptability and participation *Children level* -Engagement and acceptability of program, -Ripple effect	*School staff level* -Increased workload, -School committed staff frequent turnover, -Role of teacher’s as pseudo-parent *Parents factor* -Lack of engagement and participation, -Lack of awareness, Toothbrushes storage and hygiene issues (initial concern)	-Participants sample size issue in each of the data gathering approach -Survey didn’t include all schools -Sample size of case studies was not representative of the school population -Limited number of students participated in drawing activities due to lack of parental consent	Not given
Natapav et al. (Israel) [34]	Factors associated with continuation of supervised toothbrushing program	Early childhood setting; telephonic surveys	School Teachers	*School staff level* -Teachers’ positive attitudes (70%) -Program acceptability (96%) --Willingness to teach toothbrushing skills (85%) and enjoying teaching toothbrushing skills (20%) -Correlation between Teachers' willingness for the continuation of the program with their belief in program success (r = 0.73), acceptance of their role of teaching toothbrushing skills to children (r = 0.53), and enjoying teaching toothbrushing (r = 0.59) -Statistically significant (*p* ≤ 0.05) association between teachers’ positive attitude towards the program sustainability and conduction of toothbrushing activities daily or several times a week *Children level* -84% of teachers reported that children like to learn toothbrushing skills	*School staff level* -Teachers anticipated more barriers were associated with their unwillingness for the program continuation (r = − 0.34) -Thirty percent of teachers think its parent's role to train children in toothbrushing skills -Statistically significant (*p* ≤ 0.05) association was observed in teachers’ anticipated barriers of program sustainability and frequency of conduction of toothbrushing activities once a week or less	Not reported	Ministry of Health (MOH)
Nyandindi et al. (Tanzania) [35]	Assessment of teachers'-led factors in the oral health educational programs activities	Primary schools, surveys and interviews, teachers’ oral examination and practical exercises	Teachers	*Teacher’s level* **-**85% of teachers acknowledge the importance of health sessions in enhancing their personal hygiene	*Teacher’s factors* **-**Teachers ranked health lessons in schools moderately important after reading, writing, and mathematics subjects -Twenty-four percent of teachers had taught about diet in the class, without mentioning the association between tooth decay and diet -Most of the teachers prefer to teach toothbrushing theoretically and perceive it a parent’s responsibility to teach their kids toothbrushing skills -Teachers claimed that the association between diet and tooth decay is not part of the health lesson curriculum of grade one class -Teachers complained of insufficient material and time to teach health lessons in the overly packed class of children (mean [SD]: 65 +/− 27 students) -Eleven percent of teachers perceived the need for further training in oral health education -Only 26% of teachers had skills of making wooden toothbrush *Organisational level* -School administration rarely inquired about the health lessons conducted in schools	Not given	Not given

**Table 2:** Characteristics of the oral health promotional programs

Author, year (country)	Program type	Target group/community	Program target age	Program resource provision to schools	Toothbrushing Supervision	Program toothbrushing activities
Dimitropoulos et al. (Australia) [30]	School-based toothbrushing program	Aboriginal community	5–12 years	-At the beginning of term children were provided with a toothbrush kit (including toothpaste, toothbrush, and storage container) for in-school resources storage -Free toothpaste and toothbrushes on quarterly basis	(1) Teachers in two schools (2) Teachers and oral health aide (older student) in one school	-Once daily toothbrushing with a fluoride toothpaste at the flexibility of school timing. Usually after breakfast club or during reading activity
Yusuf et al. (England) [31]	Fluoride application and toothbrushing program	Deprived area of London	3–7 years	Not specified	Tooth champions from school staff	-Fluoride application and tooth brushing sessions at schools
Glaser-Ammann et al. (Switzerland) [32]	School Dental Health Program	Generally, in Winterthur preschools	4–5 years	Not mentioned	Schools dental care instructors	-Tooth brushing exercises with high fluoridated gels
Woodall et al. (UK) [33]	School-based toothbrushing program	Underprivileged communities of Northern England-Yorkshire and Humber region	3–5 years	-Toothbrushes, toothpaste, and toothbrush storage facility	School Staff	-School toothbrushing activities at breakfast
Natapov et al. (Israel) [34]	School-based supervised toothbrushing program	Nation-wide program in all nurseries	3–4 years	-Toothbrushing storage facility and all the necessary resources	Teachers	-Daily toothbrushing at schools
Nyandindi et al. (Tanzania) [35]	School-based oral health educational programs	Both urban and rural schools in Tanzania	7-12 years	Not specified	Teachers	-Health lessons as a part of the school curriculum -Health lessons (including oral health)1 h per week - In-school wooden toothbrush development -Teaching toothbrushing techniques

Dimitropoulos et al. [34] conducted three focus groups with schoolteachers and one with support staff. Yusuf et al. [35] conducted semi-structured interviews with dentists, school staff, and program volunteers. Woodall and colleagues collected the study data by conducting case studies in three schools (including focus groups with parents, interviews with school staff, and drawing and writing sessions with school children), three interviews were conducted with oral health promoters, and surveys were sent to 18 preschools [37]. Nayandindi et al. [39] conducted interviews and surveys with schoolteachers. Natapav et al. [38] conducted telephonic surveys with schoolteachers and Glaser-Ammann et al. [36] collected study data by conducting surveys with parents.

In the included studies, data were collected from 21 school children [37], two dentists [35], two community volunteers [35], and 114 parents [37, 39]. The aggregated number of teachers, teaching assistants, and other school staff who participated in these studies were indeterminable due to the provision of inadequate information on the number of teachers, teaching assistants, and other school staff in the study by Woodall et al. [37].

Dimitropoulos et al. [34] and Yusuf et al. [35] analysed the results by adopting a thematic analysis. Woodall et al. [37] presented the results by providing descriptive statistics of the school surveys, interpretation of children's drawings, and themes that emerged from participants' interviews and focus groups transcriptions. Glaser-Ammann et al. [36] reported parents' attitude and knowledge towards the school dental health clinics by reporting descriptive statistics and Pearson Chi-squared test at a 5% level of significance. Nyandindi et al. [39] reported the survey’s results descriptively such as frequency and percentages were reported to measure teachers' knowledge, attitude, and practice towards oral health promotion activities at primary schools. The study by Natapav et al. [38] built a linear regression model to identify predictor variables (such as the extent of confidence in program success, acknowledgment of teacher's role in teaching children to brush their teeth, and tendency to enjoy teaching toothbrushing) with teacher's willingness for the continuation of supervised toothbrushing program. However, a linear regression model was explained inadequately without discussing values of odds ratios and 95% confidence intervals and the results of the study were explained by reporting the Pearson correlation coefficient.

### Quality assessment

The quality assessment of the two studies [37, 39] that used a mixed-methods approach was ascertained using the MMAT quality assessment tool [32]. Appropriate JBI tools [33] were used for qualitative studies [34, 35] and cross-sectional studies [36, 38]. The overall quality of included studies was moderate to low as per MMAT and JBI criteria (Additional file 7: Appendix 7).

The two included studies that adopted mixed method research design [37, 39] scored moderate to low in MMAT critical appraisal tool. The study by Woodall et al. [37] had an inadequate sample size for surveys and no justification of selected sample size was provided. Furthermore, no explanation for the validity of the questionnaire used was provided. Regarding the qualitative inquiry adequate information on the congruity between study methodology and objectives, methods, and interpretation of results were provided. However, the researcher’s cultural and theoretical orientation were not outlined. In the study by Nyandindi et al. [39] a validated tool was used to conduct surveys by trained interviewers, however, no information on the interviewer's calibration was provided. In the qualitative inquiry, the philosophical perspective with the study methodology was not justified. Moreover, the authors did not provide an explicit statement on the role of the researcher in the study.

Yusuf et al. [35] and Dimitropoulos et al. [34] conducted a qualitative study. These studies scored moderately on the JBI quality assessment tool by exhibiting congruity between the study methodology, and methods, analysis, and interpretation of results. However, neither of the studies reported on the philosophical perspective of selecting a qualitative inquiry. Further, no information on the investigator's cultural and theoretical orientation, nor any explicit statement regarding the influence of the researcher on the study participants or research was provided. As these studies were evaluating the perspectives of primary school-based toothbrushing program’s stakeholders and the interviews were conducted by the same team who implemented the program, therefore the potential influence of researcher positionality [40] on the research was obvious. Hence, the overall assessment categorises the study as low quality.

The studies by Glaser-Ammann et al. [36] and Natapav et al. [38] scored low to critically low in JBI critical appraisal tool for cross-sectional studies. In the study by Natapav et al. [38] lack of congruity between the study objective, data analysis, and interpretation of the results was identified. The regression model was explained inadequately, and the results were explained by reporting correlations without referring to the significance of the findings. The response rate in both studies was 50%. Only one researcher collected information in the study by Glaser-Ammann and colleagues [36]. In the study by Natapav et al. [38], multiple trained interviewers conducted telephonic surveys. However, no information on the interviewer's calibration was provided. Both these studies did not have sufficient information on selecting the sample size nor any information on the validity of data collection tools.

#### Toothbrushing activities in early childhood settings and primary schools

The salient components of toothbrushing programs have been summarised in Table 2. The toothbrushing activities in these early childhood settings and primary schools were conducted daily [34, 37, 38], weekly (in early childhood settings and primary schools) [38, 39], or annually (early childhood settings) [36] under the supervision of teachers [34, 38, 39], teachers along with the school support staff [34], school staff [35, 37] and school dental care instructors [36]. The toothbrushing schedule varied amongst the early childhood settings and primary schools. Dimitropoulos et al. [34] reported a toothbrushing routine in primary schools in the morning hours after breakfast [34] and the study by Woodall et al. [37] reported flexible timing as per the school’s feasibility except for morning hours. However, other studies did not specify the toothbrushing session timings [35, 38, 39]. In three studies, the early childhood settings and primary schools provided toothbrush storage facilities to avoid cross-contamination [34, 37, 38].

#### Enablers for toothbrushing programs in early childhood settings and primary schools

The included studies identified the following enablers for the implementation and sustainability of toothbrushing programs in early childhood settings and primary schools.

##### Organisational-level factors

Several primary school organisational level factors were identified as enablers in the implementation of toothbrushing programs. Dimitropoulos et al. [34] reported training of primary school staff and oral health aides (Aboriginal older students from the local community) in infection control, classroom-based toothbrushing activities, capacity, and capability of school staff to modify program activities. (i.e., modification of activities to handle resource wastage), strong school leadership to establish daily toothbrushing activities, oral health aide (older students from the community) involvement in school toothbrushing supervision activities, the flexibility of the program timings, and lunchtime toothbrushing were enablers to the implementation.

Woodall et al. [37] identified having teaching support workers as the main contact point for toothbrushing program with oral health promotion staff; training of school staff; adequate information for parents to continue oral health routine at home; supportive attitude of oral health promoters in addressing logistical issues; embedding oral health in the educational curriculum and sending weekly oral hygiene updates to parents as enablers in the implementation.

Yusuf et al. [35] reported a number of effective strategies to obtain parental consent for a child’s participation in primary school-based oral health activities. These included: organisation of oral health promotion classroom sessions, approaching parents in playgrounds, active involvement of teachers, and communication with school staff, translating information in local community languages for parents, and involvement of community volunteers in the primary school-based programs. Moreover, Yusuf et al. [35] identified that the program workload should be shared among several school staff and not just the headteacher for the efficient implementation of oral health educational programs.

##### Staff-level factors

A positive attitude and acceptability of the program by school staff [34, 37–39]; teachers acknowledgment of the importance of oral health [39]; teachers accountability of their role in developing good oral hygiene routine in children [37, 38]; active participation of school headteacher in oral health program implementation [37]; involvement of school support workers in conducting toothbrushing activities [37]; and embedding toothbrushing program in the school educational curriculum [37] were identified as common enablers. Furthermore, co-designing the oral health program with teachers (i.e., timing and setting of the activities) was identified as the major contributor in the toothbrushing program continuation [38].

Natapav et al. [38] reported teachers’ willingness for the continuation of the program was correlated with their belief in the program's success (*r* = 0.73), acceptance of their role in teaching toothbrushing skills to children (*r* = 0.53) and enjoying toothbrushing activities (*r* = 0.59). Statistically significant (*p* < 0.05) difference was observed for the positive attitude of the teachers and conducting the toothbrushing activities daily or several times a week. Linear regression suggested a strong relationship (adjusted *r*^*2*^ = 0.71) between teachers' positive attitudes and program sustainability.

##### Parents-level factors

The included studies identified several parent-level factors as enablers for the implementation and sustainability of toothbrushing programs in early childhood settings and primary schools. Woodall et al. [37] noted that parents’ acceptability increased participation in early childhood settings toothbrushing sessions. Whereas, Glaser-Ammann et al. [36] reported parents acceptance increased attendance in school dental programs (72%); acknowledgment of the role of dental health instructors in teaching toothbrushing skills (25%), and providing healthy diets (20%) to children were the key facilitators in implementing toothbrushing programs. However, a non-significant correlation was observed between parents’ attendance in school dental health programs and their educational level (*p* = 0.11), country of origin (*p* = 0.07), and income (*p* = 0.07).

##### Children-level factors

The included studies identified several children-level factors as enablers for the implementation and sustainability of toothbrushing programs in early childhood settings and primary schools.

Children’s engagement and acceptability of the school-based toothbrushing programs [34, 37, 38] and continuation of a similar practice at home [37, 38], and high participation of children in school toothbrushing programs (79.2%) [35] were key identified enablers.

Woodall et al. [37] reported a “Ripple effect” i.e., children disseminated toothbrushing information delivered at schools to their family members. Further, they observed from the drawing activities of children that they gained knowledge from school-based toothbrushing programs and observing oral hygiene habits of their parents. Natapav et al. [38] noted that 84% of teachers presumed that children like to learn toothbrushing skills.

Glaser-Ammann et al. [36] while collecting parents' perspectives on toothbrushing programs concluded that 60% of parents believed that their child had benefited from the program as they brushed their teeth better and 36% reported that their child consumed healthy mid-morning snacks.

#### Barriers for toothbrushing programs in early childhood settings and primary schools

The included studies identified several barriers in the implementation of toothbrushing programs at school staff levels, school level, parents and children’s levels in early childhood settings and primary schools.

##### Organisational-level factors

The included studies identified the following school-level factors as barriers in the implementation of toothbrushing programs in early childhood settings and primary schools.

Nyandindi et al. [39] identified the lack of training for school teachers to conduct oral health education sessions and toothbrushing classroom activities, and the lack of engagement from the school administration on health lessons were major barriers in the implementation of school-based oral health programs [39].

Woodall et al. [37] concluded that lack of coordinated team approach among school staff and school committed staff frequent turnover (e.g., the staff responsible for toothbrushing program implementation at school) resulted in compromising the program’s sustainability [37]. Dimitropoulos et al. [34] identified implementing the toothbrushing activity across the school as a barrier to effective implementation of the toothbrushing program.

Yusuf et al. [35] reported that inappropriate timing of the program communication by oral health promoters; excessive workload for school staff; stringent program timelines; inadequate information transfer from headteacher to school staff; and struggle for the schools to obtain consent forms from parents were reported as major barriers in program implementation.

##### Staff-level factors

The included studies identified several school staff-level factors as barriers in the implementation and sustainability of toothbrushing programs in early childhood settings and primary schools.

Dimitropoulos et al. [34] reported concerns of primary school staff on how to incorporate a toothbrushing program in their daily routine. Whereas, the study by Yusuf et al.[35] reported the frustration among primary school staff due to internal organisational factors; the timing of program implementation; and logistical space issues.

Nyandindi et al. [39] reported staff workload; inadequate time; lack of awareness of teachers on the importance of oral health; limited availability of oral health education material; and lack of buy-in from teachers for oral health training as the main barriers in the implementation of toothbrushing programs. Teachers considered it to be the responsibility of parents to supervise their child's toothbrushing habit. Furthermore, teachers had concerns that due to insufficient educational materials and time constraints they did not teach health lessons. Only 11% of the teachers perceived the need for further training in oral health education, whereas only 26% of teachers had skills in making a wooden toothbrush.

Woodall et al. [37] reported that the coordination of teachers with oral health promoters increased their workload; teacher’s frustration in acting as a pseudo- parent; a school committed staff (e.g., the staff responsible for toothbrushing program implementation at school) frequent turnover; and change of school's head staff were identified as the major barriers in the implementation of toothbrushing programs in early childhood settings.

Natapav et al. [38] concluded 20% of teachers enjoyed teaching toothbrushing. A correlation was observed between the teachers who anticipated more barriers in school-based toothbrushing activities and their unwillingness for the sustainability of the program (*r* =  − 0.34). Thirty percent of teachers thought it was the parent’s role to teach toothbrushing to children. The teachers who reported more difficulties in the implementation of the toothbrushing program in early childhood settings were found to be less engaged in classroom toothbrushing activities (*p* < 0.05).

##### Parent-level factors

The two included studies identified several parent-level factors as barriers in the implementation of toothbrushing programs in early childhood settings.

Glaser-Ammann et al. [36] identified limited knowledge of the parents in understanding the role of kindergarten teachers in teaching toothbrushing skills to children. Similarly, in the study by Woodall et al. [37] difficulties in engaging parents and their poor attendance in toothbrushing sessions in early childhood settings were the major identified barriers in the implementation of toothbrushing programs.

##### Children-level factors

The study by Dimitropoulos et al. [34] has identified various children-level barriers for the implementation of toothbrushing programs in primary schools. The older-aged children had resistance towards the acceptability of toothbrushing programs and early morning toothbrushing. In the early phase of the program, children did not like the taste of toothpaste and imposed more challenges for program implementation. Further, infection control issues were observed due to the mishandling of toothpaste by older-aged children.

## Discussion

This review aimed to identify the key barriers and enablers to implementation and sustainability of toothbrushing programs in early childhood settings and primary schools. A total of six studies fulfilled the review eligibility criteria and were thereby included in this review. These studies were conducted in early childhood settings and primary schools with established toothbrushing activities targeting children aged 3–12 years. By adopting diverse data-collection techniques, these studies collected data from different stakeholders (including schoolteachers and staff, parents, children, health promoters, and volunteers). The key barriers and enablers were identified at an organisational, school staff, parents, and children’s level.

### Barriers and enablers at the organisational level

Evidence-based health promotional resources concluded the successful implementation and sustainability of toothbrushing programs at organisational level depends on a number of factors including integration of toothbrushing activities with other health-promoting programs; involvement of non-teaching staff in school-based activities; active engagement of peer leaders and health promoters; and inclusion of oral health information in school educational curriculum [41]. This review findings are consistent with the above-reported evidence.

Literature suggests the feasibility of integrating oral health information in school educational curriculum [42, 43] can have a positive impact on promoting oral health. However, teachers with more autonomy to alter and integrate toothbrushing activities and shape their educational innovation are more accepting of integrating oral health information in their curriculum. They can also teach with more enthusiasm and are less resistant to integrating oral health into the curriculum [44]. Moreover, school-based programs need to be instituted opportunistically [41] at a flexible timing to suit the needs of schools and educators.

Peer support may play a crucial role in improving student motivation and academic engagement by enhancing the perceived importance of oral health among students and interests in the learning tasks [45, 46]. The frequent interactions and discussions with peers provide motivation, guidance, and cognitive support which has a direct impact on students' engagement in the learning tasks [47]. The findings from this review suggest that peer involvement, such as with older students, positively impacts the implementation of toothbrushing programs in early childhood and primary school settings. These results are consistent with studies from Ireland [48] and Germany [49] on primary school children that concluded peer support was effective to promote oral health among young children. Similarly, the study by Haleem Abdul et al. [50] concluded that the peer-led approach was more effective than the teacher-led approach in improving oral health among school children. A randomised controlled trial by Vangipuram et al. [51] on the assessment and comparison of oral health education delivered by peers and dentists in school settings reported that the students in peer-led groups exhibited better oral health behaviour as compared to dentist-led groups and control groups (no intervention provided).

An alternative strategy of health promotion includes the involvement of lay health advisors (LHA) that health professionals train to promote health in the community [52, 53]. Due to personal connection with the community, LHAs are more effective in delivering medical support services to hard-to-reach populations than conventional medical health providers [54]. Literature summarises the effective role of LHA in the community awareness and education regarding communicable diseases [52, 55], maternal and child health issues [56, 57], provision of mental health counselling [58], support in other non-communicable conditions [59] and oral health promotion [60]. The findings of our review highlight the importance of community volunteers in health promotional activities conducted in school settings. Like LHAs, community volunteers have an extensive understanding of community needs, which is also helpful in developing a supportive environment and sustainability of the oral health programs [61]. Therefore, their participation in school-based oral health programs has been recommended in health-promoting activities [62].

In this review, the school-committed staff (e.g., the school staff responsible for the toothbrushing program implementation in schools) frequent turnover was identified as a key barrier in the implementation of toothbrushing activities in early childhood settings. A similar finding was reported in an Australian study, which implemented frequent toothbrushing activities with school children; however, the results were not sustained when the key school champions left the schools [63]. Moreover, school-based toothbrushing programs often encounter criticism due to infection control concerns [41]. Similar infection control issues were observed in the findings of the current review.

### Barriers and enablers at the staff-level

The teacher's role is crucial in the development of good oral health habits among children. Likewise, school staff's commitment is imperative for the continuation of school-based initiatives to promote health [64]*.* The successful implementation and sustainability of school-based oral health activities demand a high level of cooperation and buy-in from the school staff [65]. A study from Uganda concluded that a positive attitude of teachers towards the program and their enthusiasm in participation in daily toothbrushing sessions was an important aspect in the success of toothbrushing programs [66]. Moreover, the toothbrushing program in schools of Dubai concluded that the recommendation and enforcement from the school administration enhance both teacher and students' compliance with the program [67]. A review on the implementation and compliance with health policies in school settings concluded that school commitment and staff support for improving the health of children were key contributors in the implementation of health policies in educational settings [68].

School-based toothbrushing programs are heavily criticised due to barriers in implementation or sustainability imposed by a lack of support from teaching staff in educational settings [41] as observed in the studies included in this review. To reduce the burden on teachers and school staff, support mechanisms such as employing older students from the community [65] or paying hourly wages to parents have been effective for the easier implementation of toothbrushing programs in educational settings [69].

### Barriers and enablers at the parent’s-level

In this review, the included studies reported that the positive attitude of the parents, their acceptability, and increased participation were major enablers in the implementation success and sustainability of toothbrushing activities in early childhood settings and primary schools. Literature suggests school-based oral health programs are sustainable if it involves active participation by parents and linking oral health activities at home [41]. In a study from Scotland, the key element in the successful implementation of a school-based toothbrushing program was the connection between school and home environments by the provision of toothpaste, toothbrushes, toothbrushing charts, and stickers for a continuation of toothbrushing activities at home [63]. This also provided opportunities to discuss oral health issues with families and communities and may be crucial in reducing the burden of dental caries among children by encouraging toothbrushing activities at home, school, and any other settings involving children.

Scott and colleagues noted that limited knowledge of the parents on the opportunity to participate in toothbrushing programs, their perception that school and health education curricula do not permit their involvement, and the fear that their child may feel embarrassed with their participation were key factors for their limited participation in school-based health education activities [70]. Studies on human behaviour support the notion that children with parental support are less likely to experience suicidal thoughts or emotional distress, exhibit healthy dietary patterns, and are more engaged in their school [71]. In addition, studies have shown a direct relationship between parents’ active engagement in children’s school activities and quitting smoking [72] and promoting positive health behaviours among children [73]. Hence, to enhance parents’ involvement in school-based toothbrushing programs the Centre for Disease Control and Prevention has proposed the Parents for Healthy School Framework [71]. The framework provides a roadmap to schools on how to increase parents' involvement in school health promotional programs by proposing a holistic approach on how to increase connection with parents, engage them in school health activities and ensure their sustainability in school health programs [71].

### Barriers and enablers at the children-level

The findings of this review suggest children's active participation, engagement, and acceptability of school-based toothbrushing activities are key facilitators in the implementation and sustainability of early childhood settings and primary school-based toothbrushing programs. Moreover, it was observed that the children act as change agents and tend to share the information they learnt in educational settings with their families [69]. The evaluation reports of school-based supervised toothbrushing programs concluded positive attitudes of children [74] and their acceptability of after-lunch toothbrushing [75] play a crucial role in the program’s success.

This review found resistance in older-aged children towards school-based toothbrushing activities in the initial phases of program implementation. School-aged children's physical health and development vary by their developmental stages. Others’ opinions, especially peers, can easily influence children at a concrete operational stage (i.e., 7–11 years). At the formal developmental stage, children aged 11 and above actively engage in deductive reasoning and conceptualising ideas. These cognitive-developmental variations in children are also reflected in their toothbrushing habits; that is, behaviours are directly influenced by their intentions and substantially embrace mental and cognitive aspects [76]. Moreover, the literature suggests the influence of peer support in the development of good oral health behaviour in children [41, 77]. Therefore, it is pertinent that whilst planning oral health interventions for school children, researchers need to consider their developmental stage.

School-based oral health interventions have the potential to increase children's knowledge and frequency of toothbrushing [10]. However, there is growing evidence to support the limited effectiveness of school-based interventions in long-term behaviour change [78].

### Strengths and limitations

To the best of our knowledge, this is the first systematic review that has provided comprehensive information on the barriers and enablers to implementing toothbrushing programs and sustainability in early childhood settings and primary schools. The studies included in this review captured the perspectives of a variety of stakeholders, including schoolteachers and staff, parents, children, health promoters, and volunteers. An exhaustive search of five databases and a citation chasing of previously published systematic reviews and eligible studies were performed without any restriction on publication date, type, or region, allowing the capture of all relevant literature and nullifying the chance of selection bias. The qualitative assessment of the included studies was conducted using the widely recognised JBI and MMAT tools.

Despite comprehensive electronic database and citation chasing of relevant systematic reviews and eligible studies, it is possible some relevant studies missed being included in this review. The included studies were moderate to low quality as per JBI and MMAT tools. Due to the heterogenicity of included studies, a meta-analysis was not possible. Moreover, the review was restricted to studies published only in the English language, and no thesis or conference abstracts were included.

## Implications

The findings of this review indicate it would be beneficial for policymakers to mandate oral health education in early childhood settings and primary schools’ curricula. The findings recommend policymakers and researchers undertake active community engagement in designing oral health promotion programs in early childhood settings and primary schools. Furthermore, the implementation of oral health interventions and toothbrushing activities need to consider the following to ensure success in program implementation and sustainability: linking toothbrushing activities with the home environment, active engagement of all stakeholders (organisation, teachers and staff, parents, and children), and training of school teachers, integration of oral health programs with other health programs, involvement of community volunteers and peer leaders, and active involvement of oral health promoters.

This review’s findings are also beneficial for teachers to recognise their role in integrating oral health information and toothbrushing activities into their curriculum and pedagogical practices. Moreover, these findings are helpful for parents as they play a crucial role in developing health-promoting behaviours early on in their child's life and sustaining them over time. Furthermore, parents may help to motivate their children to actively participate in school-based toothbrushing activities and continue healthy oral habits at home.

## Conclusion

Early childhood settings and primary schools are good settings for the establishment of good oral health-promoting behaviours such as toothbrushing. This review highlights the key enablers and barriers of implementation and sustainability of toothbrushing programs at multiple levels—organisational, teachers and school staff, parents, and children. A positive attitude of the school staff; the flexibility of toothbrushing sessions; the involvement of community volunteers and parents were a few of the identified enablers. However, the timing of the communication of the program, inadequate transfer of information among school staff, frequent school staff turnover, lack of parental support, and teachers feeling overburdened while acting as pseudo parents were frequently reported barriers. These aspects need to be considered for the planning, implementation, and sustainability of toothbrushing programs in such settings.

## Supplementary Information

**Additional file 1.** Previous systematic review summary.

**Additional file 2.** Eligibility criteria.

**Additional file 3.** PICO framework.

**Additional file 4.** Search strategy.

**Additional file 5.** Reasons for exclusion of studies.

**Additional file 6.** PRISMA checklist.

**Additional file 7.** Quality assessment of included studies.

## Data Availability

All data generated or analysed during this study are included in this published article [and its Additional files].

## Abbreviations

AMASTAR 02: A measurement tool to assess systematic reviews 02
CFIR: Consolidated Framework for Implementation Research
EBIs: Evidence-based health interventions
EPIS: Exploration, preparation, implementation, and sustainment
IS: Implementation science
JBI: Joanna Briggs Institute
MMAT: Mixed methods appraisal tool
PICO: Population intervention comparator outcome (PICO) criteria
PRISM: Practical, robust, implementation sustainability model
PRISMA: Preferred reporting items for systematic reviews and meta-analysis
PROSPERO: International prospective register of systematic review
RE-AIM: Reach, effectiveness, adoption, implementation, and maintenance
RCT: Randomise control trial
UK: United Kingdom
