# Supplementary material for: Barriers and enablers in the implementation and sustainability of toothbrushing programs in early childhood settings and primary schools: a systematic review
Source: BMC Oral Health. 2022 Jun 18;22:242. doi: 10.1186/s12903-022-02270-7 (PMC9206278; doi:10.1186/s12903-022-02270-7)
Supplement: Supplementary file 1 — Additional file 1. Previous systematic review summary. [file 12903_2022_2270_MOESM1_ESM.docx]

**Appendix 1. Summarised assessment table of previous reviews on toothbrushing programs in early childhood settings and primary schools**

| **Reference** | **Search end date** | **Aims /Research questions** | **Included study designs** | **Databases searched** | **Age** | **Results Explained Barriers or Enablers** | **AMSTAR 2 Scoring** |
| --- | --- | --- | --- | --- | --- | --- | --- |
| **dos Santos et al, 2018 [19]** | 2017 | Effects of supervised toothbrushing on caries incidence in children and adolescents. | Randomised and quasi-randomized controlled trials | Cochran (CENTRAL/  CCTR), MEDLINE, WEB OF SCIENCE, EMBASE, LILACS and BBO, Grey literature. Current Controlled Trials and ClinicalTrials.gov. Association for Dental Research (2001–2016) and the European Organization for Caries Research (1998–2016) | <18 years of age. | Not specified | Critically low quality |
| **Shakir et al., 2021 [20]**  (Rapid review) | 2020 | To evaluate evidence of the effectiveness of school-based behavioral interventions to improve the oral health of children aged 3-18 years in a rapid review of randomized controlled trials (RCTs) | RCTs | MEDLINE via Ovid, EMBASE, The Cochrane Library (Cochrane Central Register of Controlled Trials (CENTRAL)), Web of Science (Science citation expanded), PsycINFO via Ovid, Clinical Trials.gov and the World Health Organization International Clinical Trials Registry Platform from January 2000 through December 2020 | 3-18years | No | Critically low quality |
| **Stein et al, 2018 [21]** | 2015 | The effectiveness of oral health educational actions in the school context improving oral hygiene and dental caries in schoolchildren. | RCT | Medline, Cochran (CENTRAL/  CCTR), EMBASE, LILACS | 5 to 18 years. | Not specified | Critically low quality |
| **Dickson-Swift et al, 2017 [22]**  (Scoping review) | April 2015 | What guidelines are currently available for the development of early childhood or school toothbrushing programs? | International Guidelines and tooth brushing programs | MEDLINE, PubMed, Cochrane Library, CINAHL, Embase, Wiley Online Library & google scholar | 0-12 year | Just a few sentences on the barrier | Critically low quality |

** CCTR= Central Register of Controlled Trials*
