# Supplementary material for: Barriers and enablers in the implementation and sustainability of toothbrushing programs in early childhood settings and primary schools: a systematic review
Source: BMC Oral Health. 2022 Jun 18;22:242. doi: 10.1186/s12903-022-02270-7 (PMC9206278; doi:10.1186/s12903-022-02270-7)
Supplement: Supplementary file 2 — Additional file 2. Eligibility criteria. [file 12903_2022_2270_MOESM2_ESM.docx]

**Appendix 2**. **Eligibility criteria for inclusion in the current systematic review**

| **Inclusion criteria** | **Exclusion criteria** |
| --- | --- |
| 1. Children aged 0 months to 13years or attending early childhood settings (i.e., long day-care and preschool) and primary schools. 2. Children without any systemic disease or disability 3. Oral health programs with aspects of tooth brushing component either supervised by adults or unsupervised in early childhood settings or primary schools 4. All study designs were included without any limitations. 5. Studies with outcomes including enablers or barriers in the implementation or access of toothbrushing program 6. Full-text articles available in English | 1. Studies included children more than 13-years-old and where it was difficult to specify data related to a specific age group. 2. Children with disabilities or any systemic disease 3. Tooth brushing programs are conducted in other settings such as clinics out of school care and home-based care. 4. Oral health programs without any aspect of conducting manual toothbrushing sessions. 5. Conference abstract, editorial, discussion, study protocols, commentaries, response articles, and reviews. 6. Articles published in languages other than English |
