# Supplementary material for: Barriers and enablers in the implementation and sustainability of toothbrushing programs in early childhood settings and primary schools: a systematic review
Source: BMC Oral Health. 2022 Jun 18;22:242. doi: 10.1186/s12903-022-02270-7 (PMC9206278; doi:10.1186/s12903-022-02270-7)
Supplement: Supplementary file 3 — Additional file 3. PICO framework. [file 12903_2022_2270_MOESM3_ESM.docx]

**Appendix 3. Population Intervention Comparator Outcome (PICO) Framework**

| **Terms** | **Population** | **Intervention** | **Context** |
| --- | --- | --- | --- |
| Subject Heading | CHILD, PRESCHOOL/ or Child/ | Toothbrushing/or  Oral hygiene/ | Health Knowledge, Attitudes, Practice/ or Program Development/ or Program Evaluation/ |
| Free text | ((Child* or toddler* or preschool* or primary school* or early childhood setting*)).ti or ((Child* or toddler* or preschool* or primary school*or early childhood setting*)).ab. | ((tooth or teeth or dent* or oral) adj2 (hygiene or brushing or clean* or cleans* or habit*)).ti,ab. | (barrier* or obstacle* or challenge* or perspective* or facilitator* or enabler* or impact* or effect* or implementation* or sustainabilit* or program development* or program evaluation*).ti,ab. |
