# Supplementary material for: Barriers and enablers in the implementation and sustainability of toothbrushing programs in early childhood settings and primary schools: a systematic review
Source: BMC Oral Health. 2022 Jun 18;22:242. doi: 10.1186/s12903-022-02270-7 (PMC9206278; doi:10.1186/s12903-022-02270-7)
Supplement: Supplementary file 4 — Additional file 4. Search strategy. [file 12903_2022_2270_MOESM4_ESM.docx]

**Appendix 4.**

**Table A1. MEDLINE (via Ovid) Search Strategy**

| **#** | **Query** | **Results from 17 May 2022** |
| --- | --- | --- |
| 1 | Oral Hygiene/ | 13,308 |
| 2 | Toothbrushing/ | 7,831 |
| 3 | ((tooth or teeth or dent* or oral) adj2 (hygiene or brushing or clean* or cleans* or habit*)).ti,ab. | 22,301 |
| 4 | 1 or 2 or 3 | 33,301 |
| 5 | Child, Preschool/ or Child/ | 2,001,894 |
| 6 | (Child* or toddler* or preschool* or primary school* or early childhood setting* or daycare*).ti,ab. | 1,481,755 |
| 7 | 5 or 6 | 2,516,545 |
| 8 | Health Knowledge, Attitudes, Practice/ or Program Development/ or Program Evaluation/ | 199,781 |
| 9 | (barrier* or obstacle* or challenge* or perspective* or facilitator* or enabler* or impact* or effect* or implementation* or sustainabilit* or program development* or program evaluation*).ti,ab. | 9,209,261 |
| 10 | 8 or 9 | 9,305,284 |
| 11 | 4 and 7 and 10 | 3,214 |

**Table A2. EMBASE (via Ovid) Search Strategy**

| **#** | **Query** | **Results from 17 May 2022** |
| --- | --- | --- |
| 1 | Oral Hygiene/ | 25,678 |
| 2 | Toothbrushing/ | 11,649 |
| 3 | ((tooth or teeth or dent* or oral) adj2 (hygiene or brushing or clean* or cleans* or habit*)).ti,ab. | 24,766 |
| 4 | 1 or 2 or 3 | 46,588 |
| 5 | Child, Preschool/ or Child/ | 2,287,809 |
| 6 | (Child* or toddler* or preschool* or primary school* or daycare*).ti,ab. | 2,049,301 |
| 7 | 5 or 6 | 2,970,378 |
| 8 | Health Knowledge, Attitudes, Practice/ or Program Development/ or Program Evaluation/ | 137,347 |
| 9 | (barrier* or obstacle* or challenge* or perspective* or facilitator* or enabler* or impact* or effect* or implementation* or sustainabilit* or program development* or program evaluation*).ti,ab. | 11,993,719 |
| 10 | 8 or 9 | 12,068,954 |
| 17 | 4 and 7 and 10 | 4,154 |

**Table A3. APA** **PsycInfo (EBSCOhost) Search Strategy**

| **#** | **Query** | **Limiters/Expanders** | **Last Run Via** | **Results from 17 May 2022** |
| --- | --- | --- | --- | --- |
| S1 | SU Oral Hygiene | Expanders - Apply equivalent subjects Search modes - Boolean/Phrase | Interface - EBSCOhost Research Databases Search Screen - Advanced Search Database - APA PsycInfo | 1,795 |
| S2 | SU Toothbrushing | Expanders - Apply equivalent subjects Search modes - Boolean/Phrase | Interface - EBSCOhost Research Databases Search Screen - Advanced Search Database - APA PsycInfo | 155 |
| S3 | TI ( (tooth OR teeth OR dent* OR oral) ADJ2 (hygiene OR brushing OR clean* OR cleans* OR habit*) ) OR AB ( (tooth OR teeth OR dent* OR oral) ADJ2 (hygiene OR brushing OR clean* OR cleans* OR habit*) ) | Expanders - Apply equivalent subjects Search modes - Boolean/Phrase | Interface - EBSCOhost Research Databases Search Screen - Advanced Search Database - APA PsycInfo | 1,007 |
| S4 | S1 OR S2 OR S3 | Expanders - Apply equivalent subjects Search modes - Boolean/Phrase | Interface - EBSCOhost Research Databases Search Screen - Advanced Search Database - APA PsycInfo | 1,057 |
| S5 | SU Child, Preschool or Child/ | Expanders - Apply equivalent subjects Search modes - Boolean/Phrase | Interface - EBSCOhost Research Databases Search Screen - Advanced Search Database - APA PsycInfo | 866,765 |
| S6 | TI ( (Child* OR toddler* OR preschool* OR primary school* OR daycare*) ) OR AB ( (Child* OR toddler* OR preschool* OR primary school* OR daycare*) ) | Expanders - Apply equivalent subjects Search modes - Boolean/Phrase | Interface - EBSCOhost Research Databases Search Screen - Advanced Search Database - APA PsycInfo | 730,909 |
| S7 | S5 OR S6 | Expanders - Apply equivalent subjects Search modes - Boolean/Phrase | Interface - EBSCOhost Research Databases Search Screen - Advanced Search Database - APA PsycInfo | 733,909 |
| S8 | SU Health Knowledge, Attitudes, Practice OR SU Program Development OR SU Program Evaluation | Expanders - Apply equivalent subjects Search modes - Boolean/Phrase | Interface - EBSCOhost Research Databases Search Screen - Advanced Search Database - APA PsycInfo | 104,590 |
| S9 | TI ( (barrier* OR obstacle* OR challenge* OR perspective* OR facilitator* OR enabler* OR impact* OR effect* OR implementation* OR sustainabilit* OR program development* OR program evaluation*) ) OR AB ( (barrier* OR obstacle* OR challenge* OR perspective* OR facilitator* OR enabler* OR impact* OR effect* OR implementation* OR sustainabilit* OR program development* OR program evaluation*) ) | Expanders - Apply equivalent subjects Search modes - Boolean/Phrase | Interface - EBSCOhost Research Databases Search Screen - Advanced Search Database - APA PsycInfo | 2,108,809 |
| S10 | S8 OR S9 | Expanders - Apply equivalent subjects Search modes - Boolean/Phrase | Interface - EBSCOhost Research Databases Search Screen - Advanced Search Database - APA PsycInfo | 2,111,816 |
| S11 | S4 AND S7 AND S10 | Expanders - Apply equivalent subjects Search modes - Boolean/Phrase | Interface - EBSCOhost Research Databases Search Screen - Advanced Search Database - APA PsycInfo | 386 |

**Table A4. CINAHL (EBSCOhost) Search Strategy**

| **#** | **Query** | **Limiters/Expanders** | **Last Run Via** | **Results from 17 May 2022** |
| --- | --- | --- | --- | --- |
| S1 | MH Oral Hygiene | Expanders - Apply equivalent subjects Search modes - Boolean/Phrase | Interface - EBSCOhost Research Databases Search Screen - Advanced Search Database - CINAHL Plus with Full Text | 2842 |
| S2 | MH Toothbrushing | Expanders - Apply equivalent subjects Search modes - Boolean/Phrase | Interface - EBSCOhost Research Databases Search Screen - Advanced Search Database - CINAHL Plus with Full Text | 909 |
| S3 | TI ( (tooth OR teeth OR dent* OR oral) N2 (hygiene OR brushing OR clean* or cleans* OR habit*) ) OR AB ( (tooth OR teeth OR dent* OR oral) N2 (hygiene OR brushing OR clean* OR cleans* OR habit*) ) | Expanders - Apply equivalent subjects Search modes - Boolean/Phrase | Expanders - Apply equivalent subjects Search modes - Boolean/Phrase | 9,642 |
| S4 | S1 OR S2 OR S3 | Expanders - Apply equivalent subjects Search modes - Boolean/Phrase | Expanders - Apply equivalent subjects Search modes - Boolean/Phrase | 11,638 |
| S5 | MH Child, Preschool OR Child | Expanders - Apply equivalent subjects Search modes - Boolean/Phrase | Expanders - Apply equivalent subjects Search modes - Boolean/Phrase | 1,666 |
| S6 | TI ( (Child* OR toddler* or preschool* OR primary school* OR daycare*) ) OR AB ( (Child* OR toddler* OR preschool* OR primary school* OR daycare*) ) | Expanders - Apply equivalent subjects Search modes - Boolean/Phrase | Expanders - Apply equivalent subjects Search modes - Boolean/Phrase | 533,583 |
| S7 | S5 OR S6 | Expanders - Apply equivalent subjects Search modes - Boolean/Phrase | Expanders - Apply equivalent subjects Search modes - Boolean/Phrase | 534,065 |
| S8 | MH Health Knowledge, Attitudes, Practice OR MH Program Development OR MH Program Evaluation | Expanders - Apply equivalent subjects Search modes - Boolean/Phrase | Expanders - Apply equivalent subjects Search modes - Boolean/Phrase | 24,559 |
| S9 | TI ( (barrier* OR obstacle* OR challenge* OR perspective* OR facilitator* OR enabler* OR impact* OR effect* OR implementation* OR sustainabilit* OR program development* OR program evaluation*) ) OR AB ( (barrier* OR obstacle* OR challenge* OR perspective* OR facilitator* OR enabler* OR impact* OR effect* OR implementation* OR sustainabilit* OR program development* OR program evaluation*) ) | Expanders - Apply equivalent subjects Search modes - Boolean/Phrase | Expanders - Apply equivalent subjects Search modes - Boolean/Phrase | 1,832,564 |
| S10 | S8 OR S9 | Expanders - Apply equivalent subjects Search modes - Boolean/Phrase | Expanders - Apply equivalent subjects Search modes - Boolean/Phrase | 1,842,785 |
| S11 | S4 AND S7 AND S10 | Expanders - Apply equivalent subjects Search modes - Boolean/Phrase | Expanders - Apply equivalent subjects Search modes - Boolean/Phrase | 757 |

**Table A5. Web of Science (ISI) Search Strategy**

| **#** | **Query** | **Results 17 May 2022** |
| --- | --- | --- |
| 1 | TI= ( (tooth or teeth or dent* or oral) Near/2 (hygiene or brushing or clean* or cleans* or habit*) ) OR AB= ( (tooth or teeth or dent* or oral) Near/2 (hygiene or brushing or clean* or cleans* or habit*) ) | 17,197 |
| 2 | TI = ( (Child* OR toddler* OR preschool* OR primary school* OR daycare*) ) OR AB= ( (Child* OR toddler* OR preschool* OR primary school*) ) | 1813124 |
| 3 | TI= ( (barrier* OR obstacle* OR challenge* OR perspective* OR facilitator* OR enabler* OR impact* OR effect* OR implementation* OR sustainabilit* OR program development* OR program evaluation*) ) OR AB= ( (barrier* OR obstacle* OR challenge* OR perspective* OR facilitator* OR enabler* OR impact* OR effect* OR implementation* OR sustainabilit* OR program development* OR program evaluation*) ) | 18955432 |
| 4 | #1 AND #2 AND #3 AND #4 | 1519 |
