# Supplementary material for: Barriers and enablers in the implementation and sustainability of toothbrushing programs in early childhood settings and primary schools: a systematic review
Source: BMC Oral Health. 2022 Jun 18;22:242. doi: 10.1186/s12903-022-02270-7 (PMC9206278; doi:10.1186/s12903-022-02270-7)
Supplement: Supplementary file 5 — Additional file 5. Reasons for exclusion of studies. [file 12903_2022_2270_MOESM5_ESM.docx]

**Appendix 5**. **Reasons for exclusion of studies**

| **Author** | **Project type** | **Reason for exclusion** |
| --- | --- | --- |
| Burgess-Allen et al. 2018 (UK) | School-based tooth-brushing program and fluoride varnishing program | No information on enablers and barriers have been provided |
| Finlayson et al. 2019 (US) | Early Home Visit Program | Early Head start home visit program. Not a school-based study |
| Haleem et al. 2012 (Pakistan) | Children divided into 5 groups (1) dentist led (2) teachers led (3) Peer-led (4) self-learning strategy (5) Control, group | The results focused on the oral hygiene effectiveness of the intervention. Manual tooth brushing for school children was not part of the intervention. |
| Hopper et al, 2016 (US) | School-based brushing activity with Xylitol Toothpaste | Outcome reports the effectiveness of xylitol toothpaste in reducing plague |
| Keith et al, 1977 (US) | School-Based Oral Hygiene program | The outcome explains the effectiveness of the program |
| Leanne et a, 2020, (Australia) | Parents Educational Program | Home-based educational intervention for parents |
| Ramroop et al, 2011 (West Indies) | N/A | No actual program was implemented in the schools setting |
| Macnab et al, 2010 (Uganda) | School tooth brushing and fluoride varnishing program | Results reported the program achievements. |
| Nyandindi et al, 1995 (Tanzania) | School oral health teaching training activity | Results report the effectiveness of teachers training for oral health education |
| Parke.,1986, (US) | Dental health services and oral health education | Result reports the effectiveness of the program in reducing caries. |
| Sanders et al, 2021 (Bahamas) | School-based oral health educational program, including sealant among children | Short Report- did not meet the study inclusion criteria |
| Siegal et al, 2005 (US) | Head Start Centre Oral Health Services to Children | Not a school-based study. The outcome of the study based on the dental services provided at head start centers and access issues |
| Siregar et al,2019 (India) | School-based oral health program | Results report the children's caries status, oral hygiene, and saliva condition. |
| Takeuchi et al, 2017 (Kingdom of Tonga) | Improving School-Based Oral Health Activity in the Kingdom of Tonga (one of the components of the program) | The targeted population age exceeds the study inclusion limit (5-14 years) |
| Trubey & Chestnutt, 2013 (UK) | Formative evaluation of daily supervised tooth brushing program implementation by adopting Q- Methodology | Results report participants views before the implementation of the oral health intervention program |
| Tubert-Jeannin et al, 2012 (France) | Oral Health promotion program | Results-focused on the impact of the program on the reduction of caries. No information on the program service delivery or implementation has been provided |
| Vachirarojpisan et al, 2005 (Thailand) | Dental Health Education Program | Program conducted in Health centers. |
