# Supplementary material for: Barriers and enablers in the implementation and sustainability of toothbrushing programs in early childhood settings and primary schools: a systematic review
Source: BMC Oral Health. 2022 Jun 18;22:242. doi: 10.1186/s12903-022-02270-7 (PMC9206278; doi:10.1186/s12903-022-02270-7)
Supplement: Supplementary file 7 — Additional file 7. Quality assessment of included studies. [file 12903_2022_2270_MOESM7_ESM.docx]

**Appendix 7. Quality assessment of included studies**

**Table A1. Mixed Methodology Appraisal Checklist for Mixed-Method Studies**

| **Checklist Questions** | **Mixed Method Studies** | |
| --- | --- | --- |
|  | **Woodall et al. [33]** | **Nyandindi et al. [35]** |
| 1. Is there an adequate rationale for using a mixed methods design to address the research question? | Y | N |
| 2. Are the different components of the study effectively integrated to answer the research question? | Y | Y |
| 3. Are the outputs of the integration of qualitative and quantitative components adequately interpreted? | N | N |
| 4. Are divergences and inconsistencies between quantitative and qualitative results adequately addressed? | U | N |
| 5. Is the qualitative approach appropriate to answer the research question? | Y | Y |
| 6. Is there coherence between qualitative data sources, collection, analysis, and interpretation? | Y | Y |
| 7. Is the sampling strategy relevant to address the research question? (Quantitative component) | N | Y |
| 8. Is the statistical analysis appropriate to answer the research question? (Quantitative component) | Y | N |

Y = Yes

N = No

Unclear = U

**Table A2. JBI Checklist for Qualitative Studies**

| **Checklist Questions** | **Qualitative Studies** | | |
| --- | --- | --- | --- |
|  | **Yusuf et al. [31]** | **Dimitropoulos et al. [30]** |  |
| 1. Is there congruity between the stated philosophical perspective and the research methodology? | N | N |  |
| 2. Is there congruity between the research methodology and the research question or objectives? | Y | Y |  |
| 3. Is there congruity between the research methodology and the methods used to collect data? | Y | Y |  |
| 4. Is there congruity between the research methodology and the representation and analysis of data? | Y | Y |  |
| 5. Is there congruity between the research methodology and the interpretation of results? | Y | Y |  |
| 6. Is there a statement locating the researcher culturally or theoretically? | N | N |  |
| 7. Is the influence of the researcher on the research, and vice- versa, addressed? | N | N |  |
| 8. Are participants, and their voices, adequately represented? | N | N |  |
| 9. Is the research ethical according to current criteria or, for recent studies, and is there evidence of ethical approval by an appropriate body? | N | Y |  |
| 10. Do the conclusions drawn in the research report flow from the analysis, or interpretation, of the data? | Y | Y |  |

Y = Yes

N = No

Unclear = U

**Table A3. JBI Checklist for Descriptive Cross-Sectional Studies**

| **Checklist Criteria** | **Quantitative Studies** | |
| --- | --- | --- |
|  | **Natapov et al. [34]** | **Glaser-Ammann et al. [32]** |
| 1. Was the sample frame appropriate to address the target population? | Y | Y |
| 2. Were study participants sampled in an appropriate way? | N | N |
| 3. Was the sample size adequate? | U | N |
| 4. Were the study subjects and the setting described in detail? | Y | Y |
| 5. Was the data analysis conducted with sufficient coverage of the identified sample? | Y | Y |
| 6. Were valid methods used for the identification of the condition? | N | U |
| 7. Was the condition measured in a standard, reliable way for all participants? | Y | Y |
| 8. Was there appropriate statistical analysis? | N | Y |
| 9. Was the response rate adequate, and if not, was the low response rate managed appropriately? | N | N |

Y = Yes

N = No

Unclear = U
